# Dynamic Panel Surveillance of COVID-19 Transmission in the United States to Inform Health Policy: Observational Statistical Study

**DOI:** 10.2196/21955

**Published:** 2020-10-05

**Authors:** James Francis Oehmke, Charles B Moss, Lauren Nadya Singh, Theresa Bristol Oehmke, Lori Ann Post

**Affiliations:** 1 Buehler Center for Health Policy and Economics Feinberg School of Medicine Northwestern University Chicago, IL United States; 2 Food and Resource Economics Department University of Florida Gainesville, FL United States; 3 Civil and Environmental Engineering University of California, Berkley Berkley, CA United States

**Keywords:** COVID-19, models, surveillance, reopening America, contagion, metrics, surveillance, health policy, public health

## Abstract

**Background:**

The Great COVID-19 Shutdown aimed to eliminate or slow the spread of SARS-CoV-2, the virus that causes COVID-19. The United States has no national policy, leaving states to independently implement public health guidelines that are predicated on a sustained decline in COVID-19 cases. Operationalization of “sustained decline” varies by state and county. Existing models of COVID-19 transmission rely on parameters such as case estimates or R_0_ and are dependent on intensive data collection efforts. Static statistical models do not capture all of the relevant dynamics required to measure sustained declines. Moreover, existing COVID-19 models use data that are subject to significant measurement error and contamination.

**Objective:**

This study will generate novel metrics of speed, acceleration, jerk, and 7-day lag in the speed of COVID-19 transmission using state government tallies of SARS-CoV-2 infections, including state-level dynamics of SARS-CoV-2 infections. This study provides the prototype for a global surveillance system to inform public health practice, including novel standardized metrics of COVID-19 transmission, for use in combination with traditional surveillance tools.

**Methods:**

Dynamic panel data models were estimated with the Arellano-Bond estimator using the generalized method of moments. This statistical technique allows for the control of a variety of deficiencies in the existing data. Tests of the validity of the model and statistical techniques were applied.

**Results:**

The statistical approach was validated based on the regression results, which determined recent changes in the pattern of infection. During the weeks of August 17-23 and August 24-30, 2020, there were substantial regional differences in the evolution of the US pandemic. Census regions 1 and 2 were relatively quiet with a small but significant persistence effect that remained relatively unchanged from the prior 2 weeks. Census region 3 was sensitive to the number of tests administered, with a high constant rate of cases. A weekly special analysis showed that these results were driven by states with a high number of positive test reports from universities. Census region 4 had a high constant number of cases and a significantly increased persistence effect during the week of August 24-30. This change represents an increase in the transmission model R value for that week and is consistent with a re-emergence of the pandemic.

**Conclusions:**

Reopening the United States comes with three certainties: (1) the “social” end of the pandemic and reopening are going to occur before the “medical” end even while the pandemic is growing. We need improved standardized surveillance techniques to inform leaders when it is safe to open sections of the country; (2) varying public health policies and guidelines unnecessarily result in varying degrees of transmission and outbreaks; and (3) even those states most successful in containing the pandemic continue to see a small but constant stream of new cases daily.

## Introduction

Without question, SARS-CoV-2, the novel coronavirus that causes COVID-19 [1,2], has resulted in an unprecedented pandemic in modern history with significant morbidity and mortality [3-7]. Although some countries have had success in controlling COVID-19 [8-11], others have encountered much difficulty [12-17], resulting in significant adverse outcomes [18-21]. Beyond the overall implications related to infection and death [9,11,22-29], the COVID-19 pandemic has a deleterious impact on the global economy [27,30,31], violence [32-37], mental health [38-43], and food security [44-47], and disproportionately affects vulnerable populations such as the elderly [48-52], the poor [53,54], and racial and ethnic minorities [55-61]. We must establish COVID-19 control through good policy [10,12,62-68]; unfortunately, different states have implemented various and inconsistent COVID-19 policies [67,69-76] in the absence of a national plan [77,78]. Without a COVID-19 vaccine [79-81], we need *systematic public health surveillance* [7,82-89] to inform policies and guidelines for COVID-19 control and prevention such as quarantines, social distancing, face masks, crowd control, and hygiene to prevent viral spread [90-98]. Good surveillance can safely inform our leaders when, how, and where our country can reopen [76,99-103].

According to Teutsch and Churchill [85], public health surveillance is the “systematic, ongoing assessment of the health of a community, based on the collection, interpretation, and use of health data and information. Surveillance provides information necessary for public health decision making” (pg 1). Surveillance does not rely on a single indicator; it depends on a variety of metrics to identify high-priority COVID-19 health events such as incidence, prevalence, mortality, severity, cost, preventability, and communicability [104]. We need to meet these objectives of a surveillance system to prevent infectious diseases [104]. The United States must address several public health surveillance objectives, specifically to detect outbreaks (eg, the distribution and spread of COVID-19) and evaluate control strategies [104]. A surveillance system also includes “the functional capacity for data collection and analysis, as well as the timely dissemination of data” (pg 1) [87]. To this end, our study aims to create novel, validated metrics of speed, acceleration, and jerk in COVID-19 transmission in the United States.

The Great COVID-19 Shutdown refers to the variety of “lockdown” [105] public health policies adopted by countries around the globe to prevent the further spread of COVID-19, ranging from strict and complete quarantines [106-108] to disorganized and piecemeal closures [105]. It worked in places when it was implemented properly and in a timely manner, such as China, South Korea, Singapore, and Vietnam [10,11,62,109]. Some countries eliminated COVID-19, defined as achieving zero new cases over 14 days, while others flattened the curve [64,110-113]. Governments that failed to effectively close down public movement and interactions resulted in increases in SARS-CoV-2 infections [50,114-120]. The United States had no national policy and was late in responding to the looming pandemic [105]. In fact, COVID-19 was technically classified as an epidemic by the Centers for Disease Control and Prevention when it accounted for >7.3% of all deaths in the United States. According to the National Center for Health Statistics, this was reached during the week of March 29-April 4 when COVID-19 accounted for 13.87% of all causes of death [121].

In response to the large death toll exacted by the epidemic, states independently implemented public health guidelines [14,19,70,71,122-125] regarding closures, social distancing, masks, and hand hygiene, which begs the question: when is it safe to reopen [126]? Reopening guidelines are predicated on a sustained decline in COVID-19 cases; however, operationalization of “sustained decline” varies by state [127]. Existing contagion models for COVID-19 rely on parameters such as case estimates or R_0_ and use intensive data collection efforts [128,129]. “Static” statistical models do not capture all of the relevant dynamics required to measure sustained declines [130-135]. Moreover, existing COVID-19 models use data that are subject to significant measurement error and other contaminants. Estimates of new SARS-CoV-2 infections suffer from undercounts due to asymptomatic carriers [136,137], access to testing [9,138-140], testing delays [141], testing sensitivity and specificity [142-145], and access to health care [60,146-150]. Surveillance systems and any enumeration of COVID-19 cases will err on the side of severity, meaning the most severe cases are more likely to be captured, the consequence of which is a significant undercount [71,104,130,151-156].

The conventional approach to modeling the spread of diseases such as COVID-19 is to posit an underlying contagion model and then to seek accurate direct measurement of the model parameters such as effective transmission rates or other parameters, often through labor-intensive methods relying on contact tracing to determine the spread of the virus in a sample population. For viral epidemics with an incubation period of up to 14 days, it takes weeks if not months to generate accurate parameter estimates even for simple contagion models [130]. For example, Li et al [157] provided early estimates of contagion parameters for COVID-19 using Wuhan data from contact tracing and methods developed by Lipsitch [158] but with weak statistical properties. It estimated the serial interval distribution and R_0_ from only six pairs of cases. These models also rely on underlying assumptions about immunity, common propensity for infection, well-mixed populations, etc [159]. Improvements in the models typically focus on relaxing these assumptions, for example, disaggregating the population by geography and modeling within-geography and cross-geographical personal interactions [160]. For example, Martcheva [161] provides an excellent dynamic analysis of a wide variety of contagion models and their possible dynamics. Unfortunately, the study had limited options for the statistical inference of parameter values from actual data.

In contrast, we take an empirical approach that focuses on statistical modeling of widely available empirical data such as the number of confirmed cases or the number of tests conducted that can inform estimates of the current value of critical parameters like the infection rate or reproduction rate. We explicitly recognize that the data generating process for the reported data contain an underlying contagion component, a political-economic component such as availability of accurate test kits, a social component such as how strongly people adhere to social-distancing and shelter-in-place policies, and a sometimes inaccurate data reporting process that may obscure the underlying contagion process. We therefore seek a statistical approach that can provide meaningful information despite the complex and sometimes obfuscating data generation process. Our approach is consistent with the principles of evidence-based medicine, including controlling for complex pathways that may include socioeconomic factors such as mediating variables, and policy recommendations “based on the best available knowledge, derived from diverse sources and methods” (pg S58) [162].

There are two primary advantages to this empirical approach. First, we can apply the empirical model relatively quickly to a short data set. This advantage stems from the panel nature of the model. We used US states as the cross-sectional variable, so that a week’s data from all US states provides a reasonable sample size. In addition to enabling parameter estimation early in a pandemic, using this property we tested to see if there has been a shift in the transmission or reproductive rates of the transmission process in the past week, that is, whether there is statistical evidence that the US pandemic is peaking.

The second advantage is that the approach directly measures and informs policy-relevant variables. For example, the White House issued guidance on reopening the US economy that depends on a decrease in the documented number of cases and in the proportion of positive test results over a 14-day period, among other criteria and considerations [163]. As noted above, the number and proportion of positive test results are the outcome of a data generating process that includes not just the underlying transmission process but a multitude of mediating factors as well as idiosyncrasies of the data collection and reporting process. We specifically modeled the number of positive test results in our empirical model, which provides evidence of direct use in policy dialog.

This study has two objectives: (1) to create a proof-of-concept COVID-19 surveillance system using the United States as a prototype for a global system; and (2) to validate novel surveillance metrics/techniques including speed, acceleration, and jerk to better inform public health leaders how the pandemic is spreading or changing course.

## Methods

### Overview of Methodology

First, we will provide standard surveillance metrics including new counts of SARS-CoV-2 infections, moving 7-day averages of SARS-CoV-2 infections, rates of SARS-CoV-2 infections per 100,000 population, new numbers of COVID-19 deaths, moving 7-day averages of COVID-19 deaths, and rates of COVID-19 deaths per 100,000 population plus testing and positive testing ratios. Standard surveillance metrics are useful and allow us to compare data even though standard techniques are limited to more severe cases and suffer from data contamination.

Second, to address these data limitations we will validate novel surveillance metrics of (1) speed, (2) acceleration, and (3) jerk (change in acceleration). The basic question we are trying to inform is: how are we doing this week relative to previous weeks? From a public health perspective, in the midst of a pandemic, we would like (at least) three affirmative responses: (1) there are fewer new cases per day this week than last week, (2) the number of new cases is declining from day to day, and (3) the day-to-day decline in the number of cases is even bigger this week than last week. Additionally, we would like some indicative information about significant shifts in how the pandemic is progressing — positive shifts could be the first indicators of the emergence of a new or recurrent hotspot, and positive shifts could be first indicators of successful public health policy.

This study derives indicators to inform the three questions specified in the study objective above. Next, we provide a regression-based decomposition of the indicators. While it is beyond the scope of this study to determine the underlying causes of the pandemic and its trajectory over time, we provide a decomposition into proximate contributory factors such as whether an acceleration is due to a “natural” progression of the pandemic (eg, due to an increasing infectious population) or to a shift in an underlying model parameter (eg, a parameter shift that could be associated with reopening, other health policy changes, a viral mutation, the end of summer vacation for K-12 schools, or other underlying causes). Other factors can affect acceleration by “shifting” the underlying parameters (eg, the virus can mutate to become more or less infectious, states can impose lockdowns, social pressures can encourage or discourage people from wearing masks and social distancing, etc). Therefore, we use the regression analysis to provide a decomposition of speed, acceleration, and jerk into proximate contributory factors. Finally, this study is an innovation over traditional agnostic surveillancesystems in that we go beyond presenting metrics of the transmission of COVID-19 by providing probable scenarios regarding the context in which the disease is spreading.

The COVID Tracking Project [164] compiles data from multiple state sources on the web [165]; data for the most recent 36 days were accessed from the GitHub repository [166]. After accounting for lagged and differenced regressors, this resulted in a panel of 50 states plus the District of Columbia with 29 days in each panel (n=1352). Following Oehmke et al [167], an empirical difference equation was specified in which the number of positive cases in each state at each day is a function of the prior number of cases, the level of testing, and weekly shift variables that measure whether the contagion was growing faster, at the same rate, or slower compared to the previous weeks. This resulted in a dynamic panel model that was estimated using the generalized method of moments approach by implementing the Arellano-Bond estimator in STATA/MP, version 16.1 (StataCorp LLC).

Arellano-Bond estimation of difference equations has several statistical advantages: (1) it allows for statistical examination of the model’s predictive ability and the validity of the model specification; (2) it corrects for autocorrelation and heteroscedasticity; (3) it has good properties for data with a small number of time periods and large number of states; (4) it corrects for omitted variables issues and provides a statistical test of correction validity. With these advantages, the method is applicable to ascertaining and statistically validating changes in the evolution of the pandemic within a period of one week or less, such as changes in the reproduction rate [167-174].

### Speed: New Cases Per Day

The basic indicator of the pandemic’s status on a given day is the number of new cases on that day. Since new cases per day is a rate (value per unit of time), we will adopt physics nomenclature and refer to this as the speed of the pandemic. This is consistent with heuristic descriptions of the pandemic as spreading rapidly (ie, a large number of new cases per day) or slowly (ie, a small number of new cases per day). The public health ideal is to bring the speed of the pandemic to zero.

We report the number of new cases for each state both as a number per day and as a number per 100,000 population per day (table and column references).

For mathematical formality, we write:

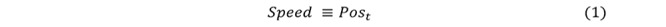



where we have suppressed the *i* subscript of the previous section. We will be reporting surveillance numbers for each state and for the District of Columbia.

### Acceleration

**Figure 1:**
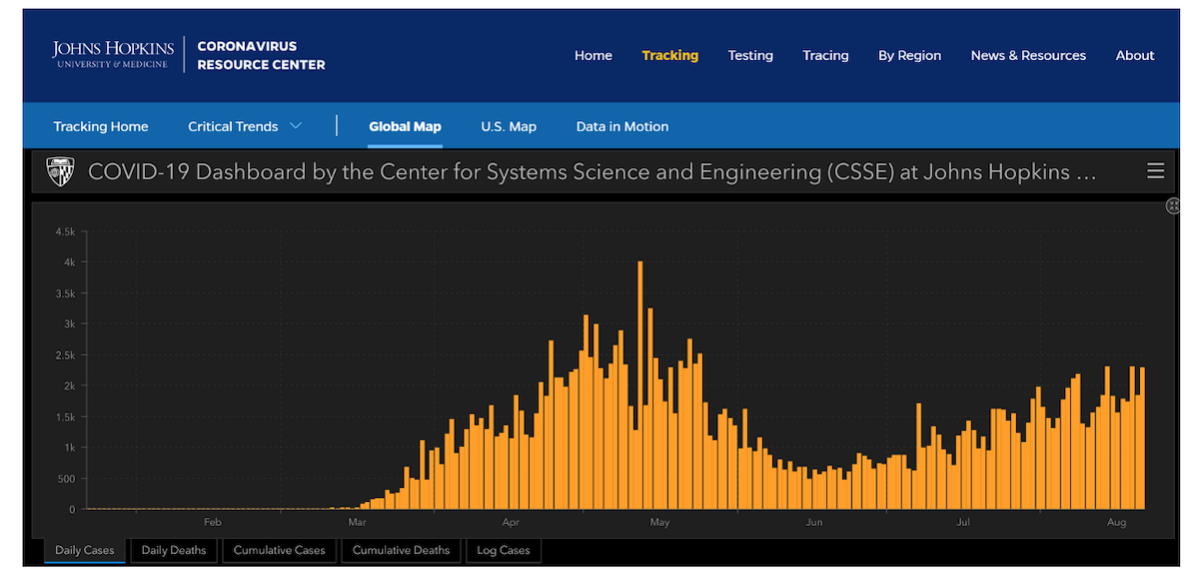
The number of positives per day in Illinois, according to the COVID-19 Dashboard of the Center for Systems Science and Engineering [175].

We are also interested in whether the number of cases per day is increasing, peaking, or decreasing, and why. Again, we will adopt physics nomenclature and refer to this datum as the acceleration. Since acceleration is difficult to ascertain on a daily basis, and there are weekend effects, etc, in the data, we report the weekly average for the acceleration as:

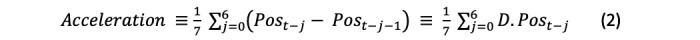



where *D.* is the difference operator. A positive acceleration indicates an increasing number of cases per day, and a negative acceleration (deceleration) indicates a decreasing number of cases per day. An acceleration of 0 is indicative of a peak, valley, or inflection point depending in part on whether the previous acceleration was positive or negative. For example, acceleration in Illinois changed from positive to zero in mid-May, indicating a peak, and from negative to zero toward the end of June, indicating a valley (Figure 1) [69,175].

We provide a regression-based decomposition of accelerations into proximate components. That is, this is the systematic component of changes in acceleration, where *t* denotes the end date for the most recent week. Subtracting Oehmke et al’s [167] equation (3) at time *t–1* from the same equation at time *t* results in:

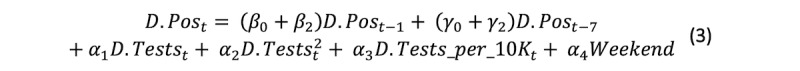



where we have suppressed the error terms and added a term for the “weekend effect.” We refer to the term containing *Pos_t_*_–1_ as the 1-day persistence effect. This, in turn, comprises a natural progression effect measured by β_0_*D.Pos_t_*_–1_ that represents the effect of a change over time in the number of new positive results where the magnitude of the effect is calibrated at the prior week’s parameter β_0_, and a shift effect β_2_*D.Pos_t_*_–1_ that measures the effect of the week’s shift in the parameter from β_0_ to β*_0_* + β_2_. The second term in this equation is the 7-day persistence effect and is analogous to the 1-day persistence effect, including its decomposition into a natural progression effect *y*_0_*D.Pos_t_*_–7_ and a shift effect *y*_2_*D.Pos_t_*_–7_. The next part of equation 2 represents the portion of acceleration that is composed of changes in the contemporaneous component of the model.

The analogous expression for 1 week prior and 2 weeks prior are:

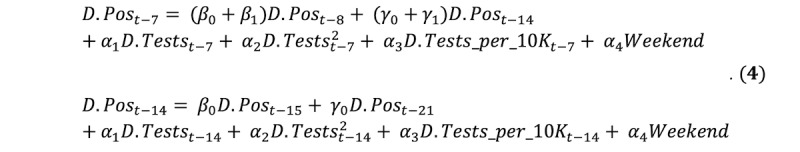



The expression for 2 weeks prior, *D.Pos_t_*_–14_, represents the baseline and does not contain any shift parameters. The shift parameters β_1_, β_2_, *y*_1_, and *y*_2_ represent shifts in the most recent 2 weeks relative to the week ending at time *t*–14.

The expressions for *D.Pos_t_* from equations 3 or 4 are easily adapted from time *t* to time *t–j* for each week and averaged over the week to provide a decomposition of acceleration as defined by equation 2.

### Jerk: The Change in Acceleration

We now address the question of whether the day-to-day increase (or decrease) in new cases the current week is bigger or smaller than the day-to-day increase (or decrease) in new cases of the past week.

Formally, for the current week we are interested in is:

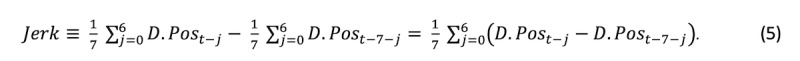



The first term to the right of the definitional equality is the average growth in the number of daily positive cases for the current week ending at time *t*, and the second term is the average increase in the number of daily positive cases for the prior week. Using physics nomenclature, the difference between these two acceleration rates is the “jerk.” A positive jerk indicates that the acceleration in the number of daily cases this week is greater than the average growth last week. Such a finding would be consistent with a scenario in which the pandemic was experiencing explosive growth; where a policy shift such as reopening had augmented the acceleration of the pandemic, possibly including a shift from deceleration to acceleration; or where a megaevent had “jerked” the acceleration upward, among other scenarios.

Using equation four, for the most recent week ending at time *t*, we can write:

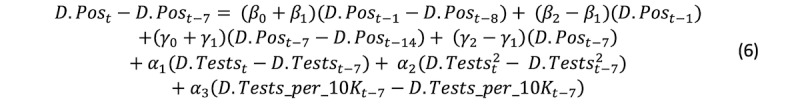



The top row contains the 1-day persistence effect’s contribution to the jerk. The first term on the right side of the equation represents the natural progression of the 1-day persistence effect on acceleration due to changes across weeks in the daily change in the number of new cases per day. The last term in the first row represents structural shifts in the 1-day persistence effect. The second row is analogous to the first row, except that it represents the 7-day persistence effect’s contribution to the jerk. The third row represents the contribution of contemporaneous effects to the jerk.

The analogous equation for the prior week is:

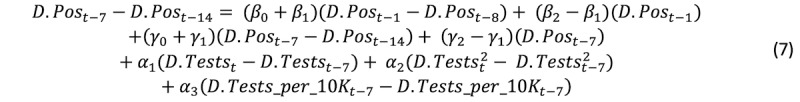



Equations 6 and 7 are easily averaged over the week to provide a decomposition of jerk as defined by equation 5.

## Results

### Regional Regression

#### Findings

We group the states according to Census region and present regression results for each region below. The biweekly surveillance products will be based on these regressions.

For Region 1 (Northeast), the regression Wald statistic shows that the model was statistically significant (*χ*^2^_10_=132, *P*<.001), and the Sargan test fails to reject the validity of the overidentifying restrictions. (*χ*^2^_252_=258, *P*=.38) (Table 1).

**Table 1:** Arellano-Bond dynamic panel data modeling of the number of daily infections reported by state, August 2-30, 2020.

Variable	Region 1		Region 2		Region 3			Region 4		
	Coefficient	*P* value	Coefficient	*P* value		Coefficient	*P* value		Coefficient	*P* value
L1Pos	0.084	.22	–0.102	.02		–0.012	.77		0.273	<.001
L1shiftAug17	–0.129	.16	0.069	.24		0.221	.02		–0.213	.03
L1shiftAug24	–0.112	.19	0.093	.10		–0.021	.84		–0.737	<.001
L7Pos	0.151	.02	0.288	<.001		0.269	<.001		0.006	.93
L7shiftAug17	–0.014	.87	–0.024	.67		–0.334	<.001		0.018	.79
L7shiftAug24	0.004	.96	0.046	.49		–0.265	.003		0.397	.02
Tests	0.003	.12	0.047	<.001		0.091	<.001		0.017	.048
Tests_squared	7.12E-09	.61	–5.05E-07	<.001		–4.89E-07	<.001		2.74E-08	.48
Tests_per_10K	1.072	.04	8.023	.002		–15.986	<.001		—^a^	—
Weekend	–14.751	.20	23.581	.28		–33.948	.58		51.977	.34
Constant	124.637	<.001	46.461	.89		429.167	<.001		397.678	<.001
Wald statistic for regression	*χ*^2^_10_=132	<.001	*χ*^2^_10_=590	<.001		*χ*^2^_10_=475	<.001		*χ*^2^_10_=316	<.001
Sargan statistic for validity	*χ*^2^_252_=258	.38	*χ*^2^_338_=373	.09		*χ*^2^_483_=446	.89		*χ*^2^_368_=370	.46

^a^Region 4 did not include the *Tests_per_100K* variable due to collinearity.

The coefficient on the first lag of the dependent variable is not statistically significant, nor are the shift parameters for the weeks of August 17 and August 24 for this coefficient. The coefficient on the 7th lag is positive and statistically significant (0.151, *P*=.02). Neither of the shift parameters for the weeks of August 17 and August 24 are statistically significant. Of the variables representing the number of tests administered, the number per 100,000 population is significant (1.072, *P*=.04). The weekend variable is not significant. The constant is positive and significant (124.637, *P*<.001).

For Region 2 (Midwest), the regression Wald statistic shows that the model was statistically significant (*χ*^2^_10_=590, *P*<.001), and the Sargan test fails to reject the validity of the overidentifying restrictions (*χ*^2^_338_=373, *P*=.09).

The coefficient on the first lag of the dependent variable is not statistically significant. The shift for the week of August 17 for this coefficient is positive and statistically significant (0.221, *P*=.02), but the shift for the week of August 24 is not significant. The coefficient on the 7th lag of the dependent variable is positive and significant (0.269, *P*<.001). Neither of the weekly shift variables for this coefficient are significant. The tests, tests squared, and tests per 10,000 population are all statistically significant (0.047, *P*<.001; –5.05E-07, *P*<.001; 8.023, *P*=.002). Neither the weekend variable nor the constant are significant.

For Region 3 (South), the regression Wald statistic shows that the model was statistically significant (*χ*^2^_10_=475, *P*<.001), and the Sargan test fails to reject the validity of the overidentifying restrictions (*χ*^2^_483_=446, *P*=.89).

The coefficient on the first lag of the dependent variable is negative and statistically significant (–0.102, *P*=.02). Neither of the weekly shift variables for this coefficient are significant. The coefficient on the 7th lag of the dependent variable is positive and significant (0.288, *P*<.001). The shifts for the weeks of August 17 and August 24 are negative and significant (–0.334, *P*<.001; and –0.265, *P*=.003, respectively). The tests, tests squared, and tests per 10,000 population are all statistically significant (0.091, *P*<.001; –4.89E-07, *P*<.001; and –15.986, *P*<.001, respectively). The weekend variable is not significant. The constant is positive and significant (429.167, *P*<.001).

For Region 4 (West), the regression Wald statistic shows that the model was statistically significant (*χ*^2^_10_=316, *P*<.001), and the Sargan test fails to reject the validity of the overidentifying restrictions (*χ*^2^_368_=370, *P*=.46).

The coefficient on the first lag of the dependent variable is negative and statistically significant (0.273, *P*<.001). The shifts for the week of August 17 and August 24 for this coefficient are negative and statistically significant (–0.213, *P*=.03; and –0.737, *P*<.001, respectively). The coefficient on the 7th lag of the dependent variable is not significant. The shift for the week of August 24 for this coefficient is positive and significant (0.397, *P*=.02), but the shift for the week of August 17 is not significant. Of the test variables, only the coefficient on the number of tests administered is significant (0.017, *P*=.048). The weekend variable is not significant. The constant is positive and significant (397.678, *P*<.001).

#### Interpretation

Region 1 appears to be fairly calm, with the only statistically significant persistence effect being a small 7-day lag effect. Region 2 is slightly less calm, but with a larger and statistically significant persistence effect and a noticeable positive effect of both the number of tests and the number of tests per 10,000. Region 3 has the largest constant (average of state-specific effects) and the largest coefficient on tests, suggesting that the number of people newly tested for the virus is an important explanatory factor for the number of new cases. Region 4 has a high constant (average state-specific value) and significant shifts in both the 1-day and 7-day persistence values.

### University Reopenings

#### Regression Results

A significant advantage of the panel data approach is that it can provide statistically valid quantifications of shifts in a fairly short period such as 1 week. Perhaps the biggest pandemic issue during the week of August 24 was the high number of cases reported on university campuses as they reopened. We address this with an additional regression analysis. Six states in Region 3 reported 500 or more cases; at least one other university in these states reported 200 or more cases (Alabama, Florida, Georgia, North Carolina, South Carolina, and Texas). To inform this university effect, we split Region 3 into two groups of states—one with a high prevalence of university COVID-19 positives (denoted as group 3a) and another comprising the remaining Region 3 states (denoted as group 3b)—and then ran the regression analysis on the two groups (Table 2).

**Table 2:** Arellano-Bond dynamic panel data modeling of the number of daily infections reported by states in Region 3, grouped by the university effect, August 2-30, 2020.

Variable	Group 3a (with university effect)			Group 3b (without university effect)	
	Coefficient	*P* value	Coefficient		*P* value
L1Pos	–0.023	.76	0.037		.34
L1shiftAug17	0.249	.13	–0.029		.74
L1shiftAug24	–0.075	.69	0.005		.95
L7Pos	0.268	<.001	0.213		<.001
L7shiftAug17	–0.364	.007	–0.100		.22
L7shiftAug24	–0.252	.12	0.092		.26
Tests	0.121	<.001	0.029		<.001
Tests_squared	–6.59E-09	<.001	–5.61E-07		<.001
Tests_per_10K	–39.704	.005	–4.402		.06
Constant	910.482	.008	245.307		<.001
Wald statistic for regression	*χ*^2^_9_=169	<.001	*χ*^2^_9_=491		<.001
Sargan statistic for validity	*χ*^2^_165_=149	.81	*χ*^2^_310_=301		.63

For each group, the Wald statistic shows that the model was statistically significant (*χ*^2^_9_=169, *P*<.001; and *χ*^2^_9_=491, *P*<.001, respectively), and the Sargan test fails to reject the validity of the overidentifying restrictions (*χ*^2^_165_=149 *P*=.81; *χ*^2^_310_=301, *P*=.63).

Without belaboring the individual coefficients, there are two important differences between the two groups. First is the coefficient on *Tests*, which numerically is the most important of the three test coefficients; group 3a (0.121, *P*<.001) is more than four times the size of the coefficient for group 3b (0.029, *P*<.001). The second important difference is that the constant for group a is more than three times the size of the constant for group b.

#### Interpretation

The larger coefficient on *Tests* means that a higher percentage of tests are associated with positive results, possibly as large as 10% for group a (considering only the linear term). The larger value of the constant (which is an average of state-specific effects) means that there are larger state-specific risk factors, possibly related to the degree of “lockdown” and social compliance with recommendations such as social distancing or wearing masks. Coupling these two effects suggests that for the week of August 24, the university effect is mostly due to increases in the number of asymptomatic students who got tested for the first time as they returned to university. This is consistent with the comparison of regional results across regions. It also suggests that the following week may be much worse if a significant fraction of the students are infectious and fail to practice social distancing, etc, thereby infecting others, who will likely show up in that week’s numbers.

These results may also help to explain spikes in other states, such as Iowa, Kansas, North Dakota, and South Dakota (which is also potentially affected by the Sturgis Motorcycle Rally), which all had significant numbers of COVID-19 cases at universities.

### Surveillance Results

Surveillance results are presented in Tables 3 and 4. The seven data elements in this proof-of-concept surveillance system are calculated as weekly averages and the speed, acceleration, and jerk are normalized per 100,000 population to compare the transmission of COVID-19 from week to week. These surveillance system data elements include (1) average weekly number of daily tests; (2) average weekly number of daily tests per 100,000 population; (3) average weekly number of daily positive tests; (4) average weekly number of daily positive tests per 100,000 population referred to as *speed;* (5) weekly average of day-to-day change in the number of positives per day per 100,000 population, referred to as acceleration; (6) change in acceleration, referred to as jerk, which is the acceleration in the current week minus the acceleration in the prior week; a sustained positive jerk is typically associated with explosive growth; and finally, (7) the 7-day lag, which is the number of new cases of COVID-19 reported today per 100,000 population (ie, today’s speed) that are associated with new cases reported 7 days ago (ie, last week’s speed), and measures how much the increase in speed from last week persists into this week. Data are presented according to US Census regions. Data element 1 is reported as a number while 2-7 are reported as a rate, which better allows for comparison between US states.

The innovation of this study is the novel metrics we derived to measure how COVID-19 is spreading and changes in terms of transmission rates. These measures should be considered in combination with traditional static numbers including transmission rates and death rates. These novel metrics measure how fast the rates are changing, accounting for their data limitations.

As an example, we tracked the transmission of COVID-19 for the state of Illinois for the week from August 17 to 23, 2020. Illinois had a weekly average of 48,181 COVID-19 tests daily, also expressed as a weekly average of 380 tests per 100,000 population per day. Illinois had a weekly average of 2026 positive tests per day. The speed of the COVID-19 transmission is measured as an increase of 15.99 persons infected per 100,000 population per day. For the week of August 17 to 23 in Illinois, COVID-19 acceleration was 0.37, which means that every day there were .37 more new cases per 100,000 than the day before, or 2.6 more cases per day per 100,000 over the course of the week. The jerk is 0.17, which means that acceleration was increasing: this increased acceleration accounted for 1.4 of the 2.6 additional cases per day per 100,000. Finally, the 7-day lag effect for speed is 3.58, which means that persistence or echo effects accounted for 3.58 or 22% of the 15.99 new daily positive cases per 100,000, which indicates an important but moderate persistence or echo effect for the week of August 17.

We see significant differences in COVID-19 transmission the following week (August 24-30, 2020). Illinois experienced a decrease in weekly average tests to 44,719 daily COVID-19 tests, also expressed as a weekly average of 353 tests per 100,000 population per day. This is 27 fewer tests per 100,000 population from last week. Illinois had a weekly average of 1923 positive tests per day, a decrease from the prior week, also expressed as a speed of 15.18 persons newly infected per day per 100,000 population. During the week of August 24-30, the acceleration decreased from the previous week to 0.11 and the jerk was negative (–0.26), indicating a leveling off of growth in new cases. Finally, the 7-day lag effect on speed is 5.35, which means that the persistence or echo effects accounted for 5.35 or over one-third of the 15.18 new daily positive cases per 100,000. The increased importance of echo effects rather than new cases from other (new) causes is consistent with a leveling off of COVID-19 growth in Illinois during the week of August 24-30.

In summary, the week of August 17-23 showed an increasing COVID-19 speed with positive acceleration and jerk. The week of August 24-30 exhibited a moderation in speed with lower acceleration and negative jerk. This is indicative of a leveling off or an inflection point: the pandemic in Illinois may be starting to decline, or this could be simply a pause before a continued increase in COVID-19 speed.

**Table 3:** Surveillance metrics for the week of August 17-23, 2020.

State		Tests per day, n (weekly average)	Daily tests per 100K people, n (daily average for the week)	Positives, n (reported number of new positive test results or confirmed cases per day per 100K people, weekly average)	Speed, n (daily positives per 100K people, weekly average)	Acceleration (day-to-day change in the number of positives per day, weekly average, per 100K people)	Jerk (week-over-week change in acceleration, per 100K people)	7-day persistence effect on speed (number of new cases per day per 100K people)
**Region 1**								
	CT	16,936	475	127	3.56	0.49	–0.16	0.32
	ME	3068	228	24	1.78	–0.06	–0.15	0.18
	MA	14,815	215	309	4.48	–0.63	–0.57	0.61
	NH	1591	117	17	1.25	0.07	0.13	0.23
	NJ	22,687	255	291	3.28	0.39	0.93	0.59
	NY	78,995	406	604	3.11	–0.03	–0.09	0.47
	PA	13,737	107	655	5.12	–0.05	0.07	0.86
	RI	5884	555	107	10.10	0.18	0.16	1.12
	VT	1273	204	6	0.96	–0.05	–0.07	0.18
**Region 2**								
	IL	48,181	380	2026	15.99	0.37	0.17	3.58
	IN	10,136	151	788	11.71	–0.26	0.38	3.41
	IA	4398	139	550	17.42	–0.43	–1.01	4.35
	KS	4654	160	594	20.40	6.63	–2.55	4.20
	MI	30,346	304	650	6.51	0.41	0.49	2.09
	MN	9467	168	633	11.23	–0.06	0.09	2.85
	MO	9888	161	1086	17.69	–0.61	–3.11	6.20
	NE	2498	129	220	11.37	–0.73	–1.56	3.89
	ND	1584	208	184	24.16	–0.06	–1.09	4.91
	OH	22035	189	931	7.96	0.03	0.35	2.40
	SD	1141	129	143	16.18	–0.24	–0.69	2.85
	WI	8511	146	708	12.17	–0.54	–0.73	3.51
**Region 3**								
	AL	10,749	219	947	19.31	–0.95	5.15	–1.33
	AR	6236	207	558	18.50	–1.41	1.77	–1.12
	DE	1637	168	63	6.51	0.18	0.44	–0.83
	DC	3313	469	53	7.49	–0.10	0.69	–0.61
	FL	28,001	130	3879	18.06	–0.54	1.09	–1.74
	GA	23,802	224	2417	22.76	–0.18	1.58	–1.77
	KY	5339	119	602	13.47	2.81	2.87	–0.89
	LA	15,107	325	718	15.44	0.13	4.65	–1.29
	MD	12927	214	556	9.19	0.14	1.09	–0.72
	MS	2015	68	823	27.64	1.18	1.88	–1.53
	NC	21,975	210	1452	13.84	0.31	0.59	–0.77
	OK	8220	208	689	17.41	0.08	–0.13	–1.11
	SC	6362	124	784	15.24	0.22	1.22	–1.08
	TN	26,836	393	1461	21.40	–0.22	0.12	–1.48
	TX	32,712	113	5994	20.67	–1.16	–2.07	–1.56
	VA	16,720	196	897	10.51	–0.07	–0.14	–0.71
	WV	5836	338	101	5.85	–0.17	0.03	–0.46
**Region 4**								
	AK	3704	506	71	9.73	–0.82	–0.94	0.28
	AZ	8414	116	652	8.96	–1.32	–1.46	0.31
	CA	106,128	269	6015	15.22	–0.40	–0.22	0.59
	CO	10,060	175	292	5.07	–0.01	0.32	0.15
	HI	2412	170	219	15.45	0.02	–0.49	0.36
	ID	2008	112	312	17.47	–0.09	2.06	0.57
	MT	1261	118	97	9.07	–0.51	–0.88	0.26
	NV	3824	124	614	19.92	–0.77	–0.24	0.57
	NM	5696	272	143	6.81	0.44	0.50	0.19
	OR	4432	105	239	5.67	–0.06	0.00	0.16
	UT	3758	117	352	10.98	–0.13	0.07	0.27
	WA	11,587	152	419	5.50	–0.17	–0.08	0.17
	WY	685	118	42	7.23	–0.57	–1.11	0.14

**Table 4:** Surveillance metrics for the week of August 24-30, 2020.

State			Tests per day, n (weekly average)		Daily tests per 100K people, n (daily average for the week)		Positives, n (reported number of new positive test results or confirmed cases per day per 100K people, weekly average)		Speed, n (daily positives per 100K people, weekly average)		Acceleration (day-to-day change in the number of positives per day, weekly average, per 100K people)		Jerk (week-over-week change in acceleration, per 100K people)		7-day persistence effect on speed (number of new cases per day per 100K people)
**Region 1**															
	CT	21,027		590		195		5.48		0.81		0.33		0.55	
	ME	3994		297		25		1.88		0.05		0.12		0.28	
	MA	24,300		353		410		5.95		0.41		1.04		0.69	
	NH	1890		139		21		1.54		–0.07		–0.15		0.19	
	NJ	26,762		301		302		3.40		0.05		–0.34		0.51	
	NY	82,233		423		623		3.20		0.09		0.12		0.48	
	PA	13,769		108		637		4.97		0.06		0.10		0.79	
	RI	4963		469		60		5.66		–1.19		–1.36		1.57	
	VT	1890		303		8		1.35		0.16		0.21		0.15	
**Region 2**															
	IL	44,719		353		1923		15.18		0.11		–0.26		5.35	
	IN	12,508		186		1054		15.66		0.56		0.82		3.92	
	IA	5017		159		921		29.18		1.95		2.38		5.83	
	KS	7366		253		838		28.78		7.63		1.00		6.82	
	MI	30,189		302		817		8.18		0.90		0.48		2.18	
	MN	8822		156		801		14.20		0.54		0.60		3.75	
	MO	8486		138		1226		19.97		1.51		2.11		5.92	
	NE	2798		145		282		14.57		1.20		1.93		3.80	
	ND	1297		170		261		34.23		1.46		1.52		8.08	
	OH	30,424		260		1066		9.12		0.35		0.32		2.66	
	SD	1270		144		292		33.04		3.86		4.10		5.41	
	WI	8464		145		728		12.50		0.22		0.76		4.07	
**Region 3**															
	AL	8485		173		1454		29.65		2.38		3.33		0.09	
	AR	6712		222		612		20.27		0.49		1.90		0.08	
	DE	1831		188		66		6.81		–0.98		–1.16		0.03	
	DC	3149		446		53		7.47		–0.45		–0.34		0.03	
	FL	24,425		114		3002		13.98		–0.26		0.28		0.08	
	GA	22,229		209		2146		20.21		–0.69		–0.51		0.10	
	KY	9483		212		643		14.40		–2.59		–5.40		0.06	
	LA	14,987		322		703		15.13		1.23		1.10		0.07	
	MD	12,335		204		527		8.72		–0.19		–0.34		0.04	
	MS	4988		168		683		22.95		0.10		–1.08		0.13	
	NC	23,543		224		1573		15.00		–0.57		–0.88		0.06	
	OK	7438		188		694		17.53		0.36		0.29		0.08	
	SC	7220		140		905		17.58		1.06		0.84		0.07	
	TN	20,545		301		1311		19.20		–2.13		–1.91		0.10	
	TX	36,669		126		4688		16.17		–0.28		0.87		0.09	
	VA	14,649		172		969		11.35		0.07		0.15		0.05	
	WV	4990		289		120		6.92		0.46		0.63		0.03	
**Region 4**															
	AK	2771		379		75		10.31		–0.25		0.57		3.92	
	AZ	6939		95		508		6.98		0.33		1.65		3.61	
	CA	98,685		250		5177		13.10		–0.26		0.14		6.14	
	CO	9257		161		308		5.35		–0.07		–0.06		2.05	
	HI	2536		179		255		17.99		0.25		0.23		6.23	
	ID	2435		136		288		16.11		0.00		0.09		7.04	
	MT	5130		480		130		12.17		0.48		0.99		3.66	
	NV	3065		100		472		15.34		–0.39		0.37		8.03	
	NM	6766		323		125		5.97		–0.48		–0.93		2.75	
	OR	4789		114		231		5.48		0.12		0.17		2.29	
	UT	4382		137		391		12.20		0.66		0.79		4.42	
	WA	11,760		154		380		4.99		1.80		1.96		2.22	
	WY	1486		257		34		5.95		0.00		0.57		2.92	

## Discussion

### Principal Findings

The dynamic panel data model is a statistically validated analysis of reported COVID-19 transmissions and an important addition to the epidemiological toolkit for understanding the progression of the pandemic. It is important to recognize that surveillance systems require a variety of metrics. Systematic surveillance with standardized measures of decreases and increases in COVID-19 transmission coupled with health policies and guidelines add a critical tool to the epidemiologic arsenal to combat COVID-19.

The specific findings of the modeling exercise confirm that SARS-CoV-2 infection rates are persistent but changeable, and for most states increasing during the period between June 13-19, 2020. We find that for every 100 new COVID-19 cases from June 13-19, the following day would result in 26 new cases, meaning there is a significant reduction each day. However, it is important to recognize that this is an average across states and that state and local experiences will vary, which we measured. From June 20-26, on average in the United States, every 100 new cases on Monday was associated with 65 new cases on Tuesday, indicating the contagion increased 2.5-fold the rate from the prior week. The American pandemic has been ramping up in the past 2 weeks.

Remarkably, the US states diverged into three distinct patterns: (1) decline, (2) constant, and (3) increases consistent with outbreaks. In the 30 states with increasing cases, over the course of 2 weeks, there was a 3.6-fold increase in new infections while the states that had sustained declines in cases decreased by 2.5-fold. Again, these are averages among the three classifications of decline, constant, and increases, but these data could be further refined to show how much each state contributed to increases and decreases. Further investigation could usefully model state and local differences in infection rates, as well as ascertain quickly whether the pandemic will continue to re-emerge in the United States, or whether infection rates will reverse track and decline again even though states reopen.

The strengths of this study are the derived new metrics of the transmission of COVID-19. The limitation of this proof-of-concept surveillance system is that it includes only dynamic cases of COVID-19 infections; a full surveillance system should also include static cases. For example, Table 2 refers only to dynamic, new infections.

Based on the empirical evidence that our metrics of the COVID-19 contagion is a good standardization of increases and decreases for public health surveillance purposes, our future work will focus on the surveillance of 195 countries in eight global regions as defined by the World Bank. When possible, we will provide subcountry-level metrics of the COVID-19 contagion beginning with US states and Canadian provinces. Our surveillance system will include estimates of speed, acceleration, and jerk in acceleration along with traditional surveillance metrics.
